# Cell death in the lateral geniculate nucleus, and its possible relationship with nicotinic receptors and sudden infant death syndrome (SIDS)

**DOI:** 10.1007/s12035-023-03332-9

**Published:** 2023-04-11

**Authors:** Cynthia Chang, Arunnjah Vivekanandarajah, Karen A Waters, Rita Machaalani

**Affiliations:** 1 Sydney Medical School, Faculty of Medicine and Health, The University of Sydney, Camperdown, NSW 2006 Australia; 2 Discipline of Medicine, Central Clinical School, Faculty of Medicine and Health, The University of Sydney, Camperdown, NSW Australia; 3 Discipline of Child and Adolescent Health, Children’s Hospital at Westmead Clinical School, Faculty of Medicine and Health, The University of Sydney, Camperdown, NSW Australia

**Keywords:** Apoptosis, Acetylcholine, Cholinergic, LGN, SUDI, Sleep

## Abstract

The role of the lateral geniculate nucleus (LGN) in vision has been extensively studied, yet its extraretinal capacities are still being investigated, including its role in arousal from sleep. The β2 nicotinic acetylcholine receptor (nAChR) subunit is involved in the laminal organisation of the LGN with magnocellular (MC) and parvocellular (PC) neurons. Sudden infant death syndrome (SIDS) occurs during a sleep period and, neuropathologically, is associated with increased neuronal cell death and altered nAChRs. A recent qualitative pilot study from our group implicates the possibility of increased neuronal death/apoptosis in the SIDS LGN. The present study used quantitative analysis to report the baseline expression of apoptotic and nAChR subunits α7 and β2 in the PC and MC layers of the LGN, to determine correlations amongst these markers within layers and across layers, and to evaluate changes in the expression of these markers in the LGN of SIDS infants, along with associations with SIDS risk factors, such as age, sex, cigarette smoke exposure, bed-sharing, and presence of an upper respiratory tract infection (URTI). Tissue was immunohistochemically stained for cell death markers of active caspase-3 (Casp-3) and TUNEL, and for the α7 and β2 nAChR subunits. Amongst 43 cases of sudden and unexpected deaths in infancy (SUDI), classifications included explained deaths (eSUDI, *n* = 9), SIDS I (*n* = 5) and SIDS II (*n* = 29). Results indicated a strong correlation of the apoptotic markers and β2 nAChR subunit between the LGN layers, but not across the markers within the layers. Amongst the diagnostic groups, compared to eSUDI, the SIDS II cases had decreased Casp-3 expression while β2 nAChR expression was increased in both PC and MC layers. Amongst the SIDS risk factors, URTI and bed-sharing were associated with changes in neuronal death but not in the α7 and β2 markers. In conclusion, our findings do not support a role for the α7 and β2 nAChRs in apoptotic regulation of the LGN layers during infancy. However, for SIDS victims, an inverse correlation between the changes for markers of apoptosis and the β2 nAChR subunit expression suggests altered LGN function.

**Supplementary Information:**

The online version contains supplementary material available at 10.1007/s12035-023-03332-9.

## Introduction

**Fig. 1:**
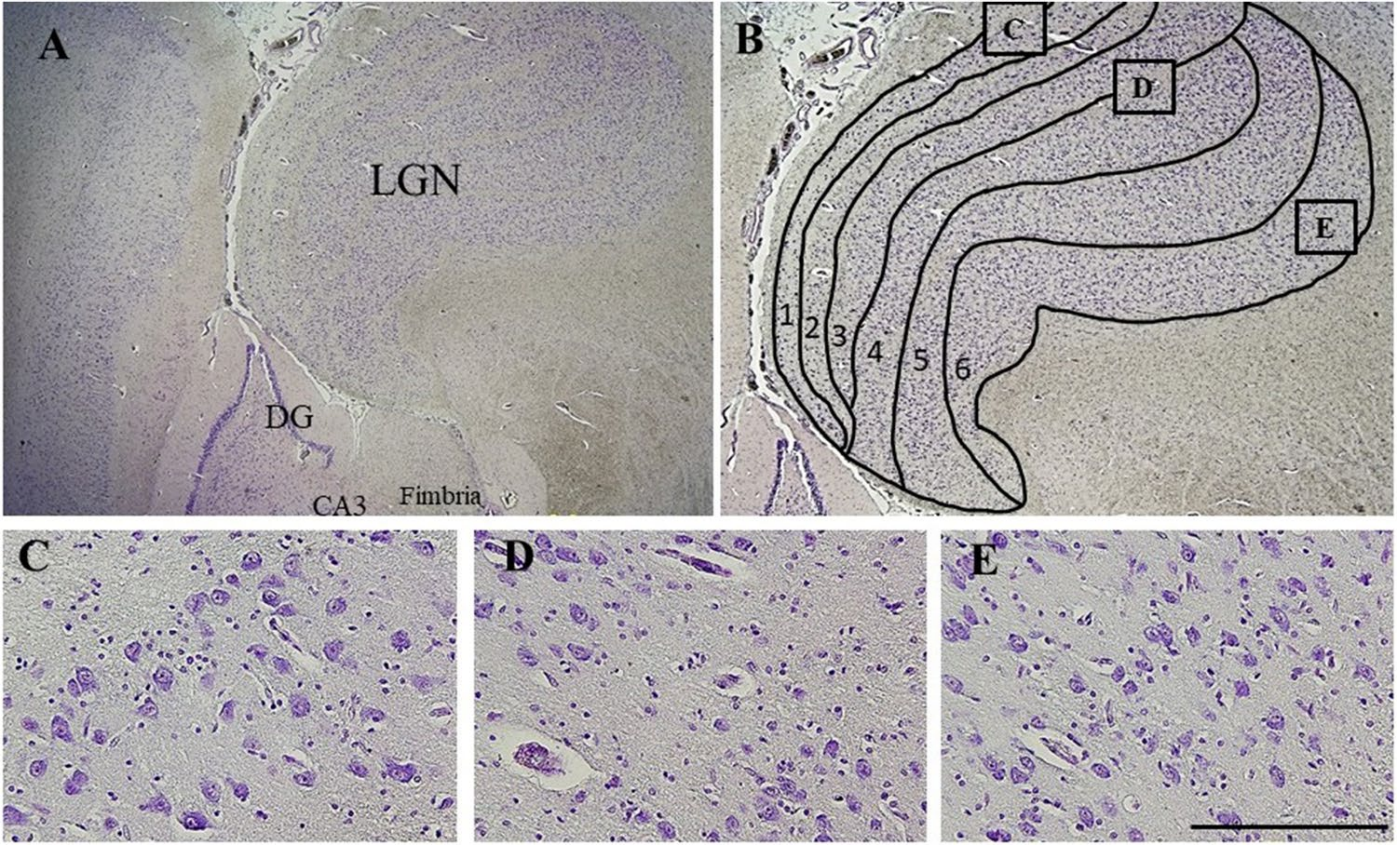
Microscopic morphology of the LGN layers and cells obtained by Cresyl Violet staining. (**A**) Micrograph illustrating the location of the LGN in coronal section at level 43 of Atlas of the Human Brain by Mai et al., 1997 and its relation to the hippocampus dentate gyrus (DG), commencement of the Cornu ammonis 3 (CA3), and fimbria. (**B**) The six laminae are indicated. 1-2 represent the magnocellular layer (MC) and 3-6 represent the parvocellular layer (PC), in between each layer are koniocellular neurons. (**C**, **D** & **E**) are magnifications of the (C) Magnocellular cells, (D) Koniocellular cells, and (E) Parvocellular cells and the boxed C & E in panel B indicate the approximate locations from where images for analysis were obtained. Scale bar represents 2.0cm for A, 1.5cm for B, and 200μm for C-E

The lateral geniculate nucleus (LGN) is located in the dorsal posterolateral thalamus, and is well known for its role in vision [1]. Yet, 80-90% of its input is from extraretinal projections [1], including the pedunculopontine tegmental nucleus which is a major source of neurons of the cholinergic ascending arousal network, and the thalamic reticular nucleus; both of which have been associated with arousal from sleep and attention [2]. Anatomically, the LGN is easily recognisable due to its distinctively laminal appearance. The LGN consists of 6 layers: 4 of parvocellular (PC) neurons and 2 of magnocellular (MC) neurons, with koniocellular neurons separating the layers (Fig. 1). The MC and PC layers are mostly studied and are physiologically, structurally and developmentally different [3]. Across species, MC neurons are described as larger than PC neurons [1, 3, 4]. Based on rodent studies, it is suggested that functionally, the MC is more closely related to visual processing with its extensive connections to the visual structures, while the PC is more related to non-visual processing with its extensive connections to the brainstem [4]. These non-visual projections have linked the LGN with a role in rapid eye movement (REM) sleep and circadian rhythms [5–7], though its specific role in non-visual processing is still being studied [3, 8].

The prevalence of sudden infant death syndrome (SIDS) has decreased since the introduction of safe sleeping programs, but the pathogenesis of the syndrome remains unclear, despite it being associated with risk factors including prematurity, male sex, cigarette smoke exposure, prone sleeping, bed-sharing and the presence of an upper respiratory tract infection [URTI] [9]. To this end, all cases of sudden unexpected deaths in infancy (SUDI) continue to be investigated by death scene investigation and autopsy, and a diagnosis of SIDS is reached only if the cause of death remains unexplained; for the explained cases, they are classified as explained SUDI (eSUDI). In 2004, the definition of SIDS was standardised to “the sudden unexpected death of an infant <1 year of age, with onset of the fatal episode apparently occurring during sleep, that remains unexplained after a thorough investigation, including performance of a complete autopsy and review of the circumstances of death and the clinical history” [10].

Abnormal cell death has been repeatedly identified in SIDS cases, affecting brain structures involved in respiratory, cardiac, autonomic and/or arousal control [11–16]. Our group recently undertook a preliminary study across 37 brain regions in SIDS evaluating two cell death markers, active caspase-3 (Casp-3) (specific for apoptosis) and Terminal deoxynucleotidyl transferase (Tdt)-mediated dUTP nick end labelling (TUNEL) [13]. The staining profile seen in the LGN was striking and deemed relevant for more detailed evaluation.

In our quest to determine the underlying mechanism(s) for increased apoptosis in the LGN, we chose to evaluate associations with the cholinergic system, given its role in apoptotic regulation [reviewed [17]]. First, cholinergic neurons are sensitive to hypoxic insults [18], and hypoxia is hypothesised to underly the pathogenesis of SIDS [19, 20]. Second, recent studies have shown decreased activity of the circulating cholinergic enzyme butyrylcholinesterase [21] and altered expression of brain nicotinic acetylcholine receptors (nAChRs) [9, 11, 12, 20, 22–25] in SIDS infants. Finally, the nAChRs regulate apoptotic expression (reviewed in [26] and correlations between markers of apoptosis and nAChR subunits, specifically α7 and β2, have been previously found in the brainstem of SIDS infants [11, 20]. Within the LGN, cholinergic activation is required for the appropriate development of the LGN neurons, with the β2 nAChR subunit particularly important for its specific anatomical and functional organisation [27, 28] and retinal afferents [29].

This study extends our recent findings of TUNEL and Casp-3 in the LGN of infants dying from SUDI [13]. In that study, a semi-quantitative scoring system was employed. Herein, we aimed to undertake a quantitative analysis of the apoptotic markers (TUNEL and Casp-3), and to include a further analysis of cholinergic receptor expression (α7 and β2 nAChR subunits) (hereafter apoptotic and cholinergic markers), in the MC and PC layers of the LGN in a larger infant cohort. The specific aims were to determine:

1. expression of the apoptotic and cholinergic markers in the PC and MC layers of the LGN in eSUDI cases,
2. relationships between the expression of apoptotic and cholinergic markers within the infant LGN,
3. whether expressions of these markers are altered in SIDS compared to eSUDI cases, and
4. any associations between known risk factors for SIDS, including cigarette smoke exposure, bed-sharing and an URTI, and the expression of apoptotic and cholinergic markers. This latter aim is important given the potential of these individual risks to induce changes in these markers, based on animal models, albeit, determined in other brain regions; cigarette smoke exposure [30, 31], bedsharing mimicked by exposure to intermittent hypercapnic hypoxia [32, 33], infections/URTI [34, 35].

## Material and Methods

### Dataset characterisation, Tissue collection, and staining

All data and brain tissue were obtained from the Department of Forensic Medicine, Glebe, NSW as part of the routine autopsy undertaken to investigate the cause of death for each infant. Cases were de-identified prior to the laboratory studies. The study was approved by the University of Sydney and the Sydney Local Health District Royal Prince Alfred hospital (RPAH) ethical committees; Protocol X13-0038 & HREC/13/RPAH/54.

Information regarding exposure to SIDS risk factors, such as cigarette smoke exposure, and demographic features, such as age at death, were collected from the forensic records. The characteristics of the dataset have been previously described, including diagnostic classification into one of the 3 groups of eSUDI, SIDS I and SIDS II, undertaken by an expert panel [14] using the criteria set by Krous et al., 2004 [10].

Tissue collection and staining were performed in our laboratory as detailed previously [32] and the same tissue sections were evaluated in our reports focused on the hippocampus in these infants [11–13, 36]. From stained tissue sections of 52 infants, a subgroup of 43 infants had tissue sections suitable for the current study based on the presence of the LGN at the correct level.

Tissue sections (7μm) mounted on 2% 3-aminopronopyltriethoxysilane-treated slides were stained by immunohistochemistry as previously detailed [11–14], using a kit for TUNEL (Millipore ApopTag Peroxidase in Situ Kit, #S7100), and antibodies for Casp-3 (Cat no: 559565; BD Pharmigen, 1:300 dilution in 1% NHS), α7 nAChR subunit (ab 10096, Abcam, 1:200 dilution in 1% NHS) and β2 nAChR subunit (sc-11372, Santa Cruz, 1:100 in 1% NHS).

### Quantitative analysis of the LGN

A Leica Upright DM6000 Light Microscope (Leica Microsystems Ltd. Heerbrugg, Switzerland), was used to ensure the presence of the LGN structure. The LGN level, size and shape were categorised based on the Atlas of the Human Brain by Mai et al., 1st Edition, 1997. Tissue within LGN levels 40-44 were used (Supplementary Figure 1) to permit differentiation of PC and MC layers. (Fig. 1).

**Fig. 2:**
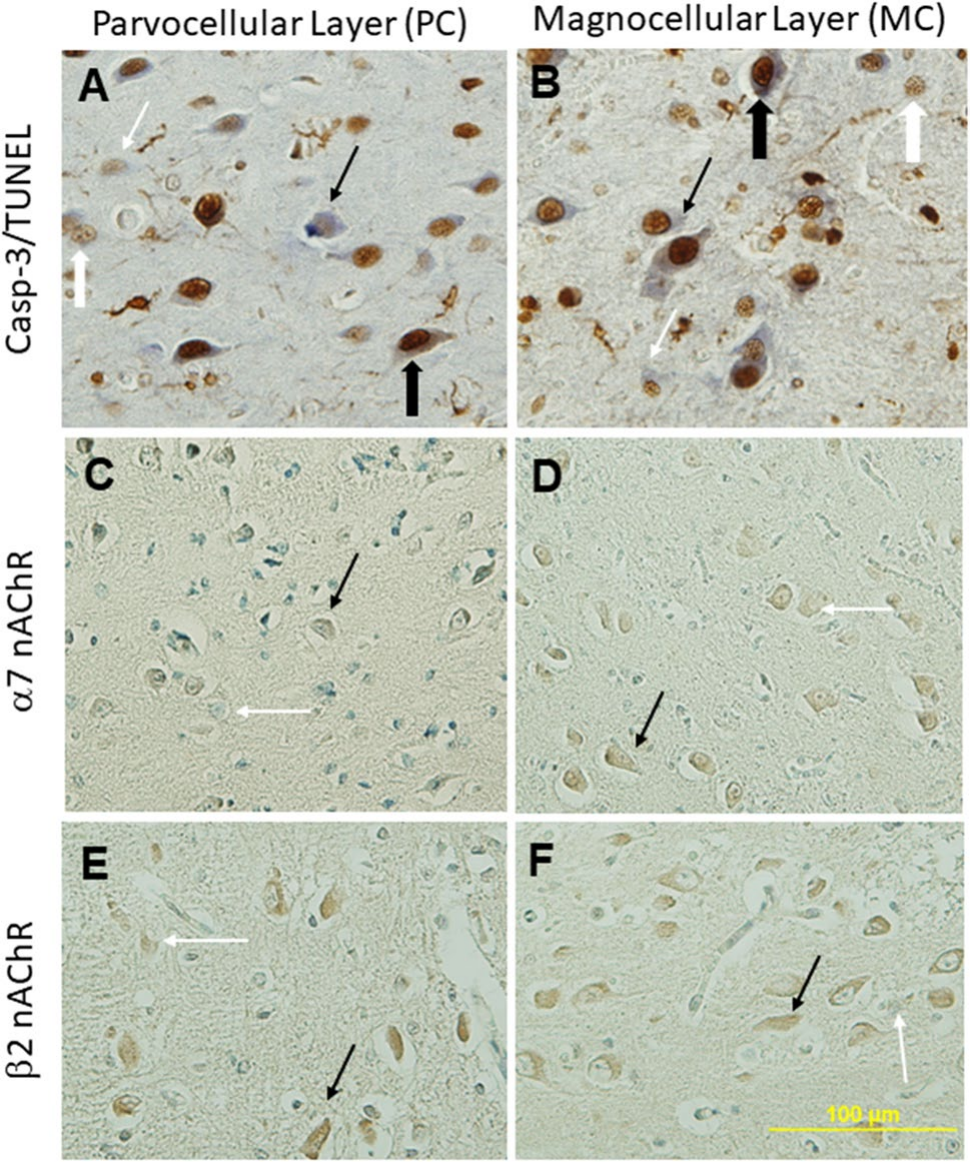
Immunostaining for the markers quantified. (**A**-**B**) TUNEL (thick arrows, positive is brown nucleus) and Casp-3 (thin arrows, positive is blue cytoplasm), (**C**-**D**) α7 nAChR subunit, and (**E**-**F**) β2 nAChR subunit. Solid black arrows represent positive neurons and white arrows represent negative neurons. Scale bar represents 100μm for all images

Quantification was conducted on four images captured using the 40X magnification lens; 2 taken within the curve from laminar 6 (PC, Fig 1 box E in panel B) and 2 from laminar 1 (MC, Fig 1 box C in panel B). A single scorer, blinded to diagnosis, counted the number of positive and negative stained neurons for all 4 markers. TUNEL and Casp-3 were visualised with brown staining in the nucleus and blue staining in the cytoplasm, respectively (Fig. 2A, B). α7 nAChR and β2 nAChR were all visualised with brown staining in the cytoplasm (Fig. 2C-F). Manual cell-counting was done using ImageJ Program (V1.51, National Institute of Health, USA). Finally, counts were converted to percentages and data are presented as % positive (of total) neurons within each layer.

For a subset of sections, intra- and inter-individual reproducibility testing was conducted by the two authors CC and AV. Intraclass correlation coefficient (ICC) analysis resulted in an inter-individual ICC of 0.72 (which is considered moderate reliability; *p* = 0.002) and an intra-individual ICC of 0.89 (considered good reliability; *p* < 0.001).

### Statistical analysis

All data was exported to Windows SPSS (Version 25; SPSS (IBM) Inc., Illinois, USA) for statistical analysis. Nominal characteristics between diagnostic groups were analysed using Chi-Square tests and expressed as percentages. Continuous data pertaining to infant characteristics were analysed using ANOVA with post-hoc least significant difference (LSD). Results are presented as mean ± standard error of the mean (SEM).

For the tissue markers, normality tests of the residuals was performed and showed a non-normal distribution for Casp-3 PC and α7 nAChR PC. As such, non-parametric tests were applied. Comparisons amongst diagnostic groups was via the Kruskal-Wallis test, and those according to the presence or absence of recognised SIDS risk factors was via the Independent samples Mann-Whitney U test. This data is presented as the median and interquartile range (Q1-Q3). Correlations between characteristics and tissue markers, and amongst the tissue markers, were analysed using Spearman's rank correlation. Significance was taken at p ≤ 0.05.

Given the growing evidence that bedsharing infants have different pathological expressions compared to non-bed-sharers [11, 15, 37–39], we included sub-analyses of bed-sharing infants from the SIDS II group.

## Results

### Data characteristics

Subgroup classifications of the 43 cases were: eSUDI (*n* = 9), SIDS I (*n* = 5) and SIDS II (*n* = 29). Within the SIDS II group, all were within the age range 1-9 months, and risks identified included neonatal or perinatal factors, including being born premature at <37w gestation (*n* = 12), had a query of possible asphyxia related to the circumstance of death, including bedsharing (*n* = 20) (Table 1), and/or based on autopsy findings as per the criteria listed in Krous et al., 2004 [10]. For the majority of these infants, more than one of these factors were present. Causes of death in the eSUDI group included infection (*n* = 5, including 3x myocarditis, 1x encephalitis, 1x bronchopneumonia+gastroenteritis), congenital syndromes (*n* = 3) and post-operative complications (*n* = 1). Clinical and autopsy characteristics did not differ amongst the diagnostic groups (Table 1). Tissue fixation time was higher in the eSUDI group (Table 1) as more cases in this group were from an older dataset (early 2000s) when the protocol for fixation time was longer and utilised 10% neutral buffered formalin (NBF) over a few weeks instead of 20% NBF over a few days [36]. Adjustment was made for the impact of fixation time on Casp-3 expression (data not shown). Risk factor profiles showed that bed-sharing and recent URTI (Table 1) were more common in SIDS II cases, as expected given its definition [10]. When the SIDS II group were separated according to bed-sharing status, associations with bed-sharing included gestational age (closer to term) and higher birth weight (Table 1).

**Table 1:** Clinical, autopsy, brain tissue and risk factor data of the study dataset amongst the 3 groups and then subdividing the SIDS II according to bed-sharing status

	eSUDI (*n *= 9)	SIDS I (*n *= 5)	SIDS II (*n *= 29)	*p-*value*	SIDS II Bed-sharers (*n *= 20)	SIDS II Non-bed-sharers (*n *= 9)	*p*-value*
Clinical data							
Birth weight (kgs)	2.5 ± 0.2	2.9 ± 0.1	3.1 ± 0.2	0.22	3.3 ± 0.2	2.6 ± 0.3	0.09
Gestational age (wks)	38.3 ± 0.7	38.8 ± 0.8	38.2 ± 0.5	0.88	39.0 ± 0.4	36.4 ± 1.0	0.05
Autopsy data							
Age at death (mths)	4.5 ± 1.0	3.0 ± 0.7	3.5 ± 0.3	0.27	3.4 ± 0.3	3.6 ± 0.6	0.73
PCA (wks)	53.4 ± 4.0	50.8 ± 2.2	52.2 ± 1.3	0.84	52.6 ± 1.4	51.6 ± 3.0	0.73
Body weight (kg)	5.7 ± 0.6	5.8 ± 1.0	6.2 ± 0.3	0.79	6.3 ± 0.4	5.8 ± 0.6	0.50
Brain weight (g)	694 ± 70	665 ± 64	732 ± 29	0.64	733 ± 33	732 ± 62	0.99
Body length (cm)	60.6 ± 2.8	61.9 ± 2.7	60.4 ± 1.5	0.92	61.0 ± 1.8	59.1 ± 2.7	0.56
HC (cm)	40.2 ± 1.5	39.6 ± 1.5	40.7 ± 0.6	0.77	40.8 ± 0.8	40.7 ± 0.9	0.98
Tissue parameters							
PMI (hrs)	25.0 ± 7.2	31.1 ± 5.8	25.5 ± 1.1	0.58	26.4 ± 1.5	23.4 ± 1.0	0.21
Fixation (wks)	3.2 ± 1.1	1.9 ± 0.3	1.2 ± 0.1	**0.01**	1.3 ± 0.2	0.9 ± 0.1	0.15
Risk factor prevalence							
Males (%)	5/9 (56)	4/5 (80)	20/29 (69)	0.62	12/20 (60)	8/9 (88)	0.20
Found prone (%)	0/4 (0)	3/5 (60)	10/27 (37)	0.17	8/18 (44)	2/9 (22)	0.24
Bed-sharing (%)	0/4 (0)	0/5 (0)	20/29 (69)	**0.01**	N/A	N/A	
Cigarette smoke exposure (%)	3/6 (50)	3/5 (60)	11/27 (41)	0.70	8/18 (44)	3/9 (33)	0.45
Recent URTI (%)	1/9 (11)	1/5 (20)	17/28 (61)	**0.02**	12/19 (63)	5/9 (55)	0.51

Results are presented as mean ± SEM. Fractions are provided to reflect instances where data was not reported

Bold used to highlight significance

*Significance taken at *p* < 0.05

PCA: post-conception age; HC: head circumference PMI: post-mortem interval. N/A: not applicable

### Baseline levels of markers and correlations with age, LGN layers and markers

In the eSUDI group, expression of apoptotic markers showed wide variation. TUNEL expression in the LGN averaged 58% (range 6%–78%), and Casp-3 averaged 43% (range 4%–70%). TUNEL averaged 58% in both the MC and PC, while Casp-3 averaged 54% and 32% in the MC and PC, respectively (medians and range provided in Table 2). For the cholinergic receptors, β2 nAChR subunit averaged 15% and α7 nAChR subunit averaged 14%, and was slightly higher in the MC than in the PC (Table 2).

**Table 2:** LGN expression of TUNEL, Caspase-3, β2 and α7 nAChR subunits for diagnostic groups

	eSUDI (*n*=9)	SIDS I (*n*=5)	SIDS II (*n*=29)	SIDS II non-bed sharers (*n*=9)	SIDS II bed-sharers (*n*=20)
TUNEL					
PC	67.5 (27.4 – 75.9)	22.4 (10.3-84.8)	37.1 (8.3-59.2)	61.6 (31.0-70.9)	23.5 (6.7-43.8)*^#^
MC	66.4 (34.2 – 73.3)	38.3 (11.6-83.7)	29.8 (10.2-67.1)	66.7 (36.7-74.0)	19.7 (7.6-46.2)*^#^
Casp-3					
PC	30.8 (11.3 – 52.9)	14.3 (0.0-32.6)	6.3 (0.0-21.0)*	0.0 (0.0-4.3)*	11.8 (0.0-25.7)*^#^
MC	58.5 (44.2 – 63.1)	45.9 (0.0-51.9)	14.5 (0.0-37.7)*	1.8 (0.0-11.2)*^	22.7 (9.6-42.5)*^#^
β2 nAChR					
PC	8.4 (5.9 – 9.9)	15.7 (12.1-22.4)	16.2 (9.7-25.6)*	25.0 (9.2-34.5)*	15.6 (9.8-23.4)*
MC	19.1 (12.5 – 22.4)	33.9 (21.3-46.0)	37.4 (28.0-47.0)*	46.3 (29.6-61.5)*	37.3 (26.5-44.2)*
α7 nAChR					
PC	9.6 (8.4 – 16.3)	13.0 (4.3-19.5)	7.1 (5.7-18.3)	6.8 (5.6-18.5)	9.8 (5.5-19.5)
MC	14.6 (10.3 – 21.9)	18.2 (4.4-28.5)	29.2 (13.6-38.6)	36.9 (17.3-53.1)	25.8 (10.9-38.5)

Results presented as median % positively stained neurons and interquartile range (Q1 – Q3)

Significance taken at *p *≤ 0.05; Bold used to highlight significance

*Significance compared to eSUDI; ^Significance compared to SIDS I; ^#^Significance compared to non-bed-sharing SIDS II

Subgroup analysis to evaluate any impact of an infectious cause of death (vs non-infectious) found no difference (*p* > 0.4 for all markers via Mann-Whitney U test - data not shown). Correlation analysis to determine whether age contributed to the wide variation in expression for TUNEL and Casp-3 showed no correlation (Supplementary Table 1).

Correlation analyses of marker expression between the PC and MC layers on the entire cohort (regardless of diagnosis classification), found the ‘within’ marker expression between the layers was positively correlated for TUNEL, Casp-3 and β2 nAChR (*p *< 0.05, Supplementary Table 2 green highlighted boxes). Analysis between the markers showed a correlation between Casp-3 and β2 nAChR expression in both PC and MC (Supplementary Table 2).

### Marker expression amongst diagnostic groups

Compared to the eSUDI group, Casp-3 was lower in both the PC (*p* = 0.014) and MC (*p* = 0.001) of SIDS II (Table 2), while β2 nAChR subunit was higher in both the PC (*p* = 0.006) and MC (*p* = 0.003) (Table 2).

Subgroup analysis of the SIDS II group according to bed-sharing status, showed that bed-sharing SIDS II infants had lower TUNEL in the PC (*p* = 0.03 vs SIDS II non-bedshare & *p* = 0.01 vs eSUDI) and the MC (*p* = 0.02 vs SIDS II non-bedshare & *p* = 0.03 vs eSUDI), and higher Casp-3 in the PC (*p* = 0.02 vs SIDS II non-bedshare & *p* < 0.001 vs eSUDI) and the MC (*p* = 0.03 vs SIDS II non-bedshare & *p* < 0.001 vs eSUDI). Lower β2 nAChR subunit expression was only seen when compared to the eSUDI group for both the PC (*p* = 0.02) and the MC (*p* = 0.01) (Table 2), with no differences in α7 nAChR subunit expression (Table 2).

The ‘within’ marker expression correlations in the PC and MC were all lost in the eSUDI group (Supplementary Table 3), but were maintained in the SIDS diagnostic sub-groups, except for β2 nAChR (Supplementary Tables 4 & 5). The between marker correlation for Casp-3 and β2 nAChR was no longer evident, however a new correlation was identified between TUNEL and α7 nAChR subunit in MC of SIDS II (Supplementary Table 5). Separating the SIDS II group according to bed-sharing status showed this new correlation to be present in the bed-sharing cohort (Supplementary Tables 6 & 7, Supplementary Figure 2).

### Associations with SIDS risk factors

Analyses for associations between marker expression and SIDS factors of URTI, cigarette smoke exposure and sex, were conducted on the whole cohort (All cases, Table 3) and then on the SIDS subset alone (combining SIDS I and SIDS II, Table 4), while the sleep related factors of being found prone and bedsharing were only conducted on the SIDS subset (Table 4).

**Table 3:** Effects of risk factors on LGN expression of TUNEL, Caspase-3, α7 and β2 nAChR subunits for ALL cases

	URTI		Smoke exposure		Sex	
	N (*n *= 23)	Y (*n *= 19)	N (*n *= 21)	Y (*n *= 17)	M (*n *= 29)	F (*n *= 14)
TUNEL						
PC	31.7 (12.5-66.1)	59.0 (8.3-68.6)	37.3 (11.5-66.2)	37.1 (8.7-67.9)	37.1 (11.8-67.9)	41.4 (7.3-63.7)
MC	40.0 (17.1-66.9)	47.7 (8.5-70.8)	26.9 (10.2-66.4)	41.7 (21.9-73.2)	41.7 (11.5-69.8)	39.4 (16.9-66.7)
Casp-3						
PC	19.5 (4.1-35.7)	7.4 (0.0-17.9)	4.8 (0.0-17.7)	15.3 (4.3-29.2)	8.6 (0.0-21.2)	11.4 (2.4-34.4)
MC	40.8 (5.3-56.3)	13.6 (0.0-35.5)	14.5 (0.0-51.9)	23.9 (9.6-49.9)	15.5 (0.0-51.8	39.9 (12.4-52.7)
β2 nAChR						
PC	9.4 (7.8-13.3)	**20.4 (15.2-25.6)**	15.3 (9.9-26.7)	14.8 (7.9-20.8)	13.9 (9.3-24.8)	14.1 (8.0-20.4)
MC	24.6 (18.6-47.0)	35.3 (25.9-45.1)	31.8 (24.4-46.8)	34.9 (19.9-43.4)	37.3 (22.4-48.0)	30.4 (20.4-41.8)
α7 nAChR						
PC	9.6 (5.6-16.8)	7.4 (6.1-24.5)	9.8 (6.2-17.3)	10.9 (5.1-21.1)	9.6 (5.7-18.1)	10.8 (6.4-16.4)
MC	22.4 (11.6-37.7)	17.3 (13.4-31.7)	22.1 (11.5-31.4)	23.0 (13.5-42.0)	22.4 (10.7-38.1)	19.8 (14.3-33.3)

Bold used to highlight significance when comparing with its counterpart at *p *≤  0.05

**Table 4:** Effects of risk factors on LGN expression of TUNEL, Casp-3, α7 and β2 nAChR subunits in SIDS subset (SIDS I and SIDS II cases combined)

	URTI		Smoke Exposure		Sex		Found prone		Bed sharing	
	N (*n *= 15)	Y (*n *= 18)	N (*n *= 18)	Y (*n *= 14)	M (*n *= 24)	F (*n *= 10)	N (*n *= 19)	Y (*n *= 13)	N (*n *= 14)	Y (*n *= 20)
TUNEL										
PC	22.4 (6.1-40.0)	56.5 (8.3-68.1)	29.8 (7.8-62.9)	33.9 (8.9-61.7)	24.3 (8.5-64.9)	39.3 (7.7-59.6)	30.8 (6.2-67.9)	40.0 (8.3-59.2)	51.9 (20.3-75.5)	**23.5** **(6.7-43.8)**
MC	29.8 (10.4-60.2)	37.3 (8.2-72.9)	23.8 (9.6-61.4)	40.5 (15.8-72.6)	34.1 (10.1-68.4)	33.2 (10.8-63.8)	38.3 (10.0-67.5)	26.9 (9.5-65.5)	63.4 (23.9-78.3)	**19.7** **(7.6-46.2)**
Casp-3										
PC	9.6 (0.0-28.0)	6.3 (0.0-14.2)	4.4 (0.0-13.4)	12.3 (1.1-24.7)	5.3 (0.0-16.7)	10.3 (0.0-26.7)	4.0 (0.0-12.3)	11.8 (0.0-27.8)	0.0 (0.0-10.3)	11.8 (0.0-25.7)
MC	22.6 (0.0-46.9)	12.7 (0.0-26.3)	13.6 (0.0-42.9)	23.3 (5.0-43.9)	12.0 (0.0-25.4)	38.8 (10.0-47.4)	12.7 (0.0-27.0)	15.5 (1.8-51.9)	3.5 (0.0-30.2)	22.7 (9.6-42.5)
β2 nAChR										
PC	11.1 (9.0-22.8)	19.9 (14.7-26.1)	16.7 (10.1-27.4)	15.7 (10.1-23.9)	15.7 (10.0-26.3)	16.7 (11.5-22.4)	18.9 (10.2-27.6)	14.7 (10.8- 24.4)	18.0 (11.7-27.6)	15.6 (9.8-23.4)
MC	36.4 (24.0-49.2)	37.3 (27.5-45.5)	37.5 (29.1-48.5)	39.7 (26.5-44.2)	38.8 (25.5-49.5)	32.5 (26.7-41.8)	33.8 (23.0-47.9)	41.0 (32.7-46.8)	43.2 (24.6-60.3)	37.3 (26.5-44.2)
α7 nAChR										
PC	9.8 (3.3-18.1)	7.1 (6.1-24.6)	7.4 (6.1-17.6)	10.9 (4.5-21.3)	9.8 (5.2-19.7)	6.8 (6.2-12.9)	10.9 (6.0-18.1)	9.8 (5.3-23.0)	7.4 (5.6-18.5)	9.8 (5.5-19.5)
MC	33.6 (18.2-38.5)	16.8 (12.2-33.4)	22.1 (9.6-33.0)	37.5 (13.6-45..4)	26.7 (13.5-38.3)	22.4 (13.5-45.7)	22.1 (14.3-37.4)	31.7 (9.3-45.8)	23.0 (16.2-37.5)	25.8 (10.9-38.5)

Results presented as median % positively stained neurons and interquartile range (Q1 – Q3) following independent samples Mann-Whitney U test

Bold used to highlight significance when comparing with its counterpart at *p *≤ 0.05

For the whole cohort, infants with an URTI had higher β2 nAChR expression in the PC layer (*p* = 0.003) (Table 3). Neither cigarette smoke exposure nor sex were associated with differences in expression levels of the apoptotic or cholinergic markers (Table 3).

For the SIDS subset, no differences were found for any of the risk factors with the exception of decreased TUNEL in both PC (*p* = 0.04) and MC (*p* = 0.03) in those found bed-sharing (Table 4).

Correlation analyses were run on the cholinergic markers to determine whether cigarette smoke exposure results in any altered relationships within the layers and/or between markers, and results showed a new correlation between Casp-3 and TUNEL in the MC with exposure (Supplementary Table 8).

### Comparisons excluding bed-sharing infants

Given the differences associated with bed-sharing, further sub-group analyses were conducted with bed-sharing infants excluded.

Analysis amongst diagnostic groups showed that compared to the eSUDI group, SIDS II infants sleeping alone (non-bed sharers) had lower Casp-3 expression in both the PC (*p* = 0.001) and the MC (*p* < 0.001) (Table 2), and higher β2 nAChR expression in the PC (*p* = 0.03) and the MC (*p* = 0.01) (Table 2). Compared to SIDS I infants, Casp-3 was lower in SIDS II non-bed-sharers in the MC only (*p* = 0.047) (Table 2).

Analysing according to the risk factors, showed that for the whole cohort, the higher β2 nAChR expression in the PC related to URTI was still maintained (*p*=0.03, Table 5 vs Table 3) but now was also associated with higher TUNEL in both the PC (*p*=0.02) and the MC (*p*=0.04) (Table 5). No differences were seen for cigarette smoke exposure and sex (Table 5).

**Table 5:** Effects of risk factors on LGN expression of TUNEL, Caspase-3, α7 and β2 nAChR subunits for ALL cases ***excluding bedsharing SIDS II***

	URTI		Smoke exposure		Sex	
	N (*n *= 16)	Y (*n *= 7)	N (*n *= 11)	Y (*n *= 9)	M (*n *= 17)	F (*n *= 6)
TUNEL						
PC	40.0 (14.6-67.9)	**73.9 (66.7-89.4)**	65.7 (21.9-73.9)	42.2 (10.9-75.3)	66.7 (18.7-75.8)	65.7 (34.0-75.3)
MC	60.2 (20.0-67.5)	**68.7 (66.2-89.3)**	66.2 (12.5-68.7)	66.7 (31.8-76.8)	66.2 (22.6-77.4)	66.7 (36.7-72.3)
Casp-3						
PC	11.4 (3.0-30.0)	0.0 (0.0-43.6)	4.0 (0.0-14.3)	15.1 (1.1-40.0)	4.3 (0.0-26.7)	21.5 (4.3-48.3)
MC	43.4 (2.6-58.2)	12.7 (0.0-51.9)	12.7 (0.0-51.8)	34.8 (0.9-53.9)	10.8 (0.0-53.9)	45.9 (18.2-64.2)
β2 nAChR						
PC	9.3 (7.2-12.9)	**22.3 (16.7-34.5)**	16.7 (10.0-27.6)	8.5 (6.7-19.5)	13.3 (8.5-26.3)	9.5 (6.7-18.5)
MC	23.5 (16.7-46.0)	30.4 (20.3-60.3)	29.6 (19.6-47.0)	21.2 (14.6-50.4)	24.6 (19.9-53.6)	26.3 (16.8-45.7)
α7 nAChR						
PC	9.1 (5.9-16.7)	9.2 (6.6-21.5)	15.1 (6.4-17.6)	10.3 (5.3-17.5)	9.0 (5.8-16.3)	12.5 (7.3-16.7)
MC	19.7 (10.7-35.3)	19.5 (15.5-36.0)	21.9 (16.8-33.6)	20.3 (12.6-41.4)	22.4 (11.6-37.1)	17.5 (15.3-20.8)

Bold used to highlight significance when comparing with its counterpart at *p *≤  0.05

For the SIDS subset, the higher β2 nAChR expression in the PC related to URTI was no longer evident, yet the higher TUNEL in both the PC (*p* = 0.008) and the MC (*p* = 0.02) were maintained (Table 6). No differences were seen for cigarette smoke exposure, sex or being found prone sleeping (Table 6).

**Table 6:** Effects of risk factors on LGN expression of TUNEL, Casp-3, α7 and β2 nAChR subunits in SIDS subset ***excluding bedsharing SIDS II***

	URTI		Smoke Exposure		Sex		Found prone	
	N (*n *= 8)	Y (*n *= 6)	N (*n *= 8)	Y (*n *= 6)	M (*n *= 12)	F (*n *= 2)	N (*n *= 9)	Y (*n *= 5)
TUNEL								
PC	22.2 (7.7-41.7)	**70.9 (65.4-90.5)**	64.1 (22.1-72.4)	41.1 (12.8-82.5)	41.1 (17.0-72.4)	70.9 (61.6-N/A)	61.6 (18.7-81.6)	40.0 (13.8-73.5)
MC	31.8 (12.5-65.7)	**74.0 (62.0-89.7)**	57.4 (12.5-76.6)	63.8 (35.1-80.8)	63.4 (21.3-76.6)	63.0 (48.1-N/A)	66.7 (31.8-79.0)	60.2 (11.6-78.6)
Casp-3								
PC	4.0 (0.0-14.3)	0.0 (0.0-15.6)	0.0 (0.0-5.8)	4.3 (0.0-32.6)	0.0 (0.0-6.3)	10.8 (0.0-10.8)	0.0 (0.0-5.8)	4.3 (0.0-17.9)
MC	3.5 (0.0-45.9)	6.3 (0.0-23.9)	3.5 (0.0-14.1)	3.5 (0.0-48.9)	0.0 (0.0-14.5)	29.3 (12.7-29.3)	3.5 (0.0-14.1)	3.5 (0.0-48.9)
β2 nAChR								
PC	13.3 (10.4-25.7)	21.5 (14.3-37.5)	20.8 (12.1-32.8)	18.0 (9.2-24.9)	18.0 (10.4-31.1)	20.3 (16.7-20.3) (7.2)	22.8 (14.3-37.5)	13.3 (10.4-24.4)
MC	43.2 (27.1-59.5)	38.3 (23.3-60.6)	38.3 (25.8-57.0)	43.2 (20.3-72.1)	46.3 (24.4-60.9)	36.8 (30.4-36.8)	30.0 (23.3-60.6)	46.3 (33.9-60.0)
α7 nAChR								
PC	10.3 (3.9-19.1)	7.4 (6.4-25.1)	7.4 (5.9-18.5)	8.3 (4.8-18.4) (16.4)	9.2 (5.3-19.1)	6.8 (6.8-6.8)^**&**^	7.4 (5.9-18.5)	9.8 (2.7-33.2)
MC	33.6 (17.6-41.4)	17.3 (14.8-47.9	21.7 (16.2-36.9)	30.2 (15.8-49.2)	26.7 (15.5-41.4)	17.3 (17.3-17.3)^**&**^	21.7 (16.2-36.9)	41.7 (8.6-68.8)

Results presented as median % positively stained neurons and interquartile range (Q1 – Q3) following independent samples Mann-Whitney U test

Bold used to highlight significance when comparing with its counterpart at *p* ≤ 0.05

^&^Only accounts for one case as staining for α7 nAChR was missing for one of the cases

## Discussion

This is the first study to provide detailed evaluation of the expression of both apoptotic and cholinergic markers in the human infant LGN. While expression of the apoptotic and β2 nAChR cholinergic markers showed good correlation across the 2 layers of the LGN, the only correlation amongst markers was between Casp-3 and β2. Interestingly, all these correlations were contingent on whether infants were bed-sharing. We discuss a potential role for the cholinergic system in development, function, and the regulation of neuronal apoptosis in the infant LGN, and of the factors identified as influencing these associations, including SIDS II classification, a history of bed-sharing and presence of an URTI at the time of death

### Tissue markers in the infant LGN

We found a wide variation in the proportion of LGN neurons undergoing apoptosis in this infant dataset with an average of 50%, and this was not correlated with age (1-9 months), regardless of the infants’ diagnosed cause for death. The 50% for TUNEL correlates with our previous qualitative analysis [13] yet the proportion for Casp-3 was higher herein. The advantages of the current study include our specific focus on the LGN, the larger study cohort, and that the quantitative analysis was undertaken separately for each layer [13]. Neurons, particularly those of the visual system, require programmed cell death to become fully developed after birth [40–42]. The presence of neuronal apoptosis in the LGN during a critical period of development has been seen in other studies and in different animal models, with apoptotic expression reducing with increasing age [42–45]. However, it is important to note that other studies report the mean number of apoptotic neurons [42, 43] rather than the percentage of positive neurons as we have done, thus it is not possible to determine comparative expression levels. Moreover, the data can vary pending on the marker being studied, as we have shown herein with the lack of any correlation ‘across’ TUNEL and Casp-3 in both layers, indicating both Casp-3 dependent and independent pathways are likely present [46]. Regardless, our data provide supporting evidence of ongoing apoptotic processes in the human infant LGN, and that the levels do not vary within the first 9 months of life but rather, do so due to other factors as will be discussed below.

Our finding of an average of 14-15% in the α7 and β2 nAChR subunits was lower than anticipated, especially for the β2 nAChR subunit which plays a key role in the functional organisation of the LGN [27–29] and for which amongst other subunits, was found to be the predominant one expressed, although α7 was not analysed in that study [29]. We did not find any other reports of the α7 nAChR in the LGN, but based on the findings that the α7 nAChR knock-out mouse has poor visual acuity due to changes in the visual cortex [47], it is reasonable to think that this receptor subunit also plays a key role in the LGN, thus warranting further studies.

Expression of our markers in the MC relative to the PC was slightly higher, consistent with current research suggesting that the MC develops and matures faster than the PC [42, 48, 49]. However, this did not affect the correlation in the expression of the markers between the layers which showed consistent distribution across the layers (internal ‘within’ correlation), particularly for the apoptotic markers.

### Comparison between diagnostic groups

In both layers of the LGN, Casp-3 expression was lower in SIDS II compared to the eSUDI group, and it was markedly lower in SIDS II infants who slept alone. This contrasts with our previous finding of increased Casp-3 expression in other brain regions in SIDS [11, 13–15]. In the absence of any changes in TUNEL expression, this may be due to the high incidence of infection/inflammatory-related deaths in our eSUDI group, with systemic injury contributing to increased markers of the early stages of apoptosis in the LGN. This has been previously seen for Casp-3 [34] and TUNEL [35] in different brain structures of rodent models of neonatal systemic inflammation, with the Casp-3 data showing it to only be increased after gram negative inflammation [34]. We postulate that this supports activation of non-Casp-3 apoptotic pathways, especially given that the eSUDI group showed no differences between the infectious and non-infectious groups. This explanation is also consistent with our finding that only TUNEL expression was increased in association with URTI, and not Casp-3, regardless of the infant’s final diagnosis. An alternative explanation could be that a protective mechanism is activated in the SIDS LGN, preventing the activation of the Casp-3- mediated apoptotic pathway. This possibility is supported by the higher β2 nAChR expression we found in SIDS II cases, since β2 nAChR has a neuroprotective role (reviewed in [50]) that can become activated during hypoxia-induced apoptosis [18].

Our data showed that both layers were affected equally in SIDS II, and is contrary to the literature indicating functional differences in response to insult [51] with data indicating the MC layer is more susceptible, for example when exposed to alcohol in the third trimester in monkeys [52], and in neurodegenerative diseases, including Alzheimer’s and Parkinson’s (reviewed in [53]). It is possible that age, and the duration and type of insult, may contribute to these differences.

Given the importance of β2 nAChR and Casp-3, independent from apoptosis, in the development of the LGN, we hypothesise that the differences in marker expression described in SIDS cases could also reflect an insufficient LGN development, leading to dysfunction. Though we could not assess the function of the LGN, we speculate that the differences seen have implications for its roles in both visual and higher-order processing, including arousal from sleep [5–7]. In the LGN, the orexinergic system plays a role in arousal from sleep, and this non-visual role of the LGN has been linked to control of REM sleep and the circadian rhythm [7, 54, 55]. In light of our previous finding of decreased orexin expression in the SIDS hypothalamus and nuclei of the pons [56], we speculate that the same system could be disrupted in the LGN and contributing to an arousal deficit.

### Effects of risk factors

Bed-sharing and the presence of an URTI, were the only risk factors associated with changes in marker expression.

Bed-sharers had reduced expression of TUNEL in both PC and MC, suggesting reduced cell death in the LGN of this group. We consider the impact of bed-sharing to most likely relate to hypoxia, and SIDS II bed-sharers showed differences in all markers except for the α7 nAChR, in both layers of the LGN. Current literature recognises the effect of hypoxia on the LGN (reviewed in [57]); however, we found no literature on the effect of hypoxia on nAChR or apoptotic expression in the LGN, so further investigation into this relationship is warranted. The LGN has great capability in adaptive reorganisation following a mild traumatic brain injury [58], and the increase in Casp-3 and decrease in TUNEL and β2 nAChR expression might reflect this.

When analysing excluding the bed-sharers, the presence of URTI was the only risk factor associated with changes: increased TUNEL in both layers, and an increase in β2 nAChR in the PC. An association between URTI and TUNEL has been previously described by our group with the hypothesis that a systemic immune challenge leads to neuronal cell death [11, 13]. The increase of β2 nAChR in the PC could represent the unique laminal organisation of the LGN, its connections to specific brain structures or its response to injury. One connection of interest is the locus coeruleus (LC) from the noradrenergic ascending arousal network that is associated with respiratory control [59] and converges onto the PC layer of the LGN [4, 60]. Given that a disruption of the LC’s noradrenergic fibres can result in tight-junction disorganisation and a leaky blood brain barrier [61], thought to be responsible, for example, in the similar levels of circulating mercury between the PC LGN and LC in human adults [60], the increase in β2 nAChR expression in the PC associated with URTI could be an up-stream effect via the LC, especially as a moderate level of β2 nAChR expression has been previously recognised in the infant LC [12, 62]. Unfortunately, in our previous studies of the LC [12, 62, 63] we did not evaluate the effects of URTI, and thus, a direct link between this risk factor and marker expression in the LC remains unknown.

### Limitations

A main limitation of the study is that we were not able to perform stereological based quantification. This was due to the study being retrospective in nature with limited tissue available to us after coronial post-mortem investigation. As such, we cannot report on the uniformity of the changes observed throughout the LGN, nor if they are due to changes in neuron numbers. However, our method of %positivity allows for comparison amongst cases where limited tissue is available for analysis.

## Conclusion

In a cohort of SUDI cases, we found an average of 50% of neurons in the infant LGN undergoing apoptosis. Associations with α7 and β2 nAChR subunit expression were weak, suggesting that the apoptotic process observed is not regulated by α7 nor β2 nAChRs. Changes seen in SIDS II infants and in sub-groups exposed to various SIDS risk factors support a neuroprotective role of the β2 nAChRs. Variations we found amongst the groups with exposure to different SIDS risk factors support current literature suggesting that the LGN is sensitive to insults such as hypoxia and infection, rather than being attributable to any specific cause of SIDS.

## Supplementary information

ESM 1 (PDF 590 kb)

## Data Availability

The data of this study are available from the corresponding author upon reasonable request.

## Abbreviations

ACh: Acetylcholine
Casp-3: Caspase 3
LC: Locus coeruleus
LGN: Lateral Geniculate Nucleus
LSD: Least Significant Difference
MC: Magnocellular
nAChR: Nicotinic acetylcholine receptor
PC: Parvocellular
SEM: Standard Error of the Mean
SIDS: Sudden Infant Death Syndrome
SUDI: Sudden Unexpected Death in Infancy
TUNEL: Terminal Deoxynucleotidyl Transferase (Tdt)-Mediated dUTP Nick End
REM: Rapid Eye Movement sleep
URTI: Upper Respiratory Tract Infection
