# Supplementary material for: Cell death in the lateral geniculate nucleus, and its possible relationship with nicotinic receptors and sudden infant death syndrome (SIDS)
Source: Mol Neurobiol. 2023 Apr 11;60(7):4120–31. doi: 10.1007/s12035-023-03332-9 (PMC10224858; doi:10.1007/s12035-023-03332-9)
Supplement: Supplementary file 1 — (PDF 590 kb) [file 12035_2023_3332_MOESM1_ESM.pdf]

Supplementary Data

Table 1. Spearman's rank correlations between cell death markers and age in the whole cohort, and then based on diagnosis.

	All cases	eSUDI only	SIDS I only	SIDS II only
TUNEL PC	r = -0.117<br>p = 0.460	r = -0.095<br>p = 0.823	r = -0.500<br>p = 0.391	r = -0.130<br>p = 0.501
TUNEL MC	r = -0.209<br>p = 0.185	r = -0.048<br>p = 0.910	r = -0.800<br>p = 0.104	r = -0.190<br>p = 0.324
Casp-3 MC	r = 0.001<br>p = 0.997	r = 0.167<br>p = 0.693	r = -0.564<br>p = 0.322	r = 0.001<br>p = 0.994
Casp-3 PC	r = 0.047<br>p = 0.771	r = 0.000<br>p = 1.000	r = -0.462<br>p = 0.434	r = -0.008<br>p = 0.969

eSUDI: explained sudden unexpected death in infancy; SIDS: sudden infant death syndrome; PC: parvocellular layer; MC: magnocellular layer

Tables 2- 9. Correlation outputs between the markers using Spearman's rank correlation. Green highlighting indicates statistical significance with p<0.05 for the correlations of relevance, i.e. the same marker was present across the 2 layers, and/or different markers within the same layer.

Table 2. All cases (n=42)

	TUNEL PC	TUNEL MC	Casp PC	Casp MC	Beta PC	Beta MC	Alpha PC	Alpha MC
TUNEL PC								
TUNEL MC	r = 0.887<br>p < 0.001							
Casp PC	r = 0.307<br>p = 0.054	r = 0.221<br>p = 0.171						
Casp MC	r = 0.373<br>p = 0.018	r = 0.257<br>p = 0.109	r = 0.867<br>p < 0.001					
Beta PC	r = 0.024<br>p = 0.886	r = -0.097<br>p = 0.570	r = -0.333<br>p = 0.047	r = -0.396<br>p = 0.017				
Beta MC	r = -0.251<br>p = 0.133	r = -0.263<br>p = 0.116	r = -0.266<br>p = 0.116	r = -0.335<br>p = 0.046	r = 0.339<br>p = 0.037			
Alpha PC	r = 0.136<br>p = 0.442	r = 0.195<br>p = 0.270	r = -0.099<br>p = 0.582	r = -0.093<br>p = 0.606	r = 0.298<br>p = 0.097	r = -0.107<br>p = 0.558		
Alpha MC	r = -0.007<br>p = 0.968	r = 0.193<br>p = 0.282	r = -0.147<br>p = 0.421	r = -0.251<br>p = 0.167	r = 0.148<br>p = 0.427	r = 0.204<br>p = 0.271	r = 0.282<br>p = 0.107	

Table 3. eSUDI only (n=8)

	TUNEL PC	TUNEL MC	Casp PC	Casp MC	Beta PC	Beta MC	Alpha PC	Alpha MC
TUNEL PC								
TUNEL MC	r = 0.635<br>p = 0.091							
Casp PC	r = 0.548<br>p = 0.160	r = 0.323<br>p = 0.435						
Casp MC	r = 0.905<br>p = 0.002	r = 0.575<br>p = 0.136	r = 0.667<br>p = 0.071					
Beta PC	r = -0.214<br>p = 0.645	r = -0.468<br>p = 0.289	r = 0.321<br>p = 0.482	r = -0.286<br>p = 0.535				
Beta MC	r = 0.321<br>p = 0.482	r = 0.000<br>p = 1.000	r = -0.107<br>p = 0.819	r = 0.214<br>p = 0.645	r = -0.310<br>p = 0.456			
Alpha PC	r = -0.371<br>p = 0.468	r = 0.058<br>p = 0.913	r = 0.600<br>p = 0.208	r = 0.029<br>p = 0.957	r = 0.486<br>p = 0.329	r = -0.600<br>p = 0.208		
Alpha MC	r = -0.200<br>p = 0.704	r = -0.638<br>p = 0.173	r = 0.943<br>p = 0.005	r = 0.029<br>p = 0.957	r = 0.829<br>p = 0.042	r = -0.143<br>p = 0.787	r = 0.714<br>p = 0.071	

Table 4. SIDS I only (n=5)

	TUNEL PC	TUNEL MC	Casp PC	Casp MC	Beta PC	Beta MC	Alpha PC	Alpha MC
TUNEL PC								
TUNEL MC	r = 0.900<br>p = 0.037							
Casp PC	r = 0.667<br>p = 0.219	r = 0.667<br>p = 0.219						
Casp MC	r = 0.359<br>p = 0.553	r = 0.359<br>p = 0.553	r = 0.895<br>p = 0.040					
Beta PC	r = 0.800<br>p = 0.200	r =0.800<br>p = 0.200	r = 0.600<br>p = 0.400	r = 0.000<br>p = 1.000				
Beta MC	r = -0.800<br>p = 0.200	r = -0.800<br>p = 0.200	r = -0.400<br>p = 0.600	r = -0.200<br>p = 0.800	r = -0.400<br>p = 0.600			
Alpha PC	r = 0.200<br>p = 0.800	r = 0.400<br>p = 0.600	r = -0.738<br>p = 0.262	r = -0.738<br>p = 0.262	r = 0.500<br>p = 0.667	r = -0.500<br>p = 0.667		
Alpha MC	r = -0.800<br>p = 0.200	r = -0.400<br>p = 0.600	r = 0.211<br>p = 0789	r = 0.211<br>p = 0.789	r = -0.500<br>p = 0.667	r = 0.500<br>p = 0.667	r = -0.400<br>p = 0.600	

Table 5. SIDS II only (n=29)

	TUNEL PC	TUNEL MC	Casp PC	Casp MC	Beta PC	Beta MC	Alpha PC	Alpha MC
TUNEL PC								
TUNEL MC	r = 0.860 p < 0.001							
Casp PC	r = -0.036 p = 0.857	r = -0.048 p = 0.812						
Casp MC	r = 0.095 p = 0.638	r = 0.055 p = 0.786	r = 0.840 p < 0.001					
Beta PC	r = 0.160 p = 0.434	r = -0.026 p = 0.901	r = -0.381 p = 0.060#	r = -0.335 p = 0.102				
Beta MC	r = -0.077 p = 0.710	r = -0.132 p = 0.520	r = -0.098 p=0.640	r = -0.082 p = 0.695	r = 0.244 p = 0.229			
Alpha PC	r = 0.088 p = 0.683	r = 0.102 p = 0.636	r = -0.238 p = 0.273	r = -0.151 p = 0.492	r = 0.404 p = 0.056	r = 0.114 p = 0.606		
Alpha MC	r = 0.343 p = 0.109	r = 0.565 p = 0.005#	r = -0.080 p = 0.722	r = -0.105 p = 0.642	r = -0.019 p = 0.934	r = 0.092 p = 0.684	r = 0.372 p = 0.081	

#Based on the next 2 tables, this is due to the bed-sharing cohort. Refer to Supplement Fig 2.

Table 6. SIDS II bed-sharers (n=20)

	TUNEL PC	TUNEL MC	Casp PC	Casp MC	Beta PC	Beta MC	Alpha PC	Alpha MC
TUNEL PC								
TUNEL MC	r = 0.857 p < 0.001							
Casp PC	r = 0.244 p = 0.313	r = 0.274 p = 0.255						
Casp MC	r = 0.447 p = 0.055	r = 0.450 p = 0.053	r = 0.709 p < 0.001					
Beta PC	r = -0.006 p = 0.980	r = -0.143 p = 0.559	r = -0.464 p = 0.052	r = -0.423 p = 0.080				
Beta MC	r = -0.0455 p = 0.051	r = -0.465 p = 0.045	r = -0.051 p = 0.840	r = 0.102 p = 0.686	r = 0.063 p = 0.797			
Alpha PC	r = 0.070 p = 0.790	r = 0.151 p = 0.563	r = -0.236 p = 0.379	r = -0.162 p = 0.548	r = 0.453 p = 0.068	r = 0.265 p = 0.305		
Alpha MC	r = 0.521 p = 0.039	r = 0.676 p = 0.004	r = 0.079 p = 0.779	r = 0.114 p = 0.685	r =-0.059 p = 0.829	r = -0.068 p = 0.803	r = 0.391 p = 0.134	

Table 7. SIDS II non-bed-sharers (n=9)

	TUNEL PC	TUNEL MC	Casp PC	Casp MC	Beta PC	Beta MC	Alpha PC	Alpha MC
TUNEL PC								
TUNEL MC	r = 0.800 p = 0.010							
Casp PC	r = -0.027 p = 0.949	r = -0.273 p = 0.513						
Casp MC	r = -0.025 p = 0.952	r = -0.444 p = 0.271	r = 0.697 p = 0.054					
Beta PC	r = 0.036 p = 0.939	r = -0.214 p = 0.645	r = 0.355 p = 0.435	r = 0.371 p = 0.413				
Beta MC	r = -0.107 p = 0.819	r = -0.107 p = 0.819	r = 0.571 p = 0.180	r = 0.222 p = 0.632	r = 0.357 p = 0.432			
Alpha PC	r = 0.321 p = 0.482	r = 0.214 p = 0.645	r = -0.394 p = 0.382	r = -0.185 p = 0.691	r = 0.143 p = 0.787	r = -0.600 p = 0.020		
Alpha MC	r = -0.393 p = 0.383	r = 0.000 p = 1.000	r = -0.256 p = 0.579	r = -0.704 p = 0.077	r = -0.257 p = 0.623	r = 0.143 p = 0.787	r = 0.107 p = 0.819	

Table 8. All cases with cigarette smoke exposure (n=17)

	TUNEL PC	TUNEL MC	Casp PC	Casp MC	Beta PC	Beta MC	Alpha PC	Alpha MC
TUNEL PC								
TUNEL MC	r = 0.882<br>p < 0.001							
Casp PC	r = 0.499<br>p = 0.058	r = 0.310<br>p = 0.260						
Casp MC	r = 0.631<br>p = 0.012	r = 0.536<br>p = 0.039	r = 0.885<br>p < 0.001					
Beta PC	r = 0.279<br>p = 0.334	r = 0.332<br>p = 0.246	r = -0.281<br>p = 0.353	r = -0.275<br>p = 0.364				
Beta MC	r = -0.275<br>p = 0.342	r = -0.240<br>p = 0.409	r = -0.308<br>p = 0.306	r = -0.374<br>p = 0.209	r = 0.059<br>p = 0.840			
Alpha PC	r = 0.396<br>p = 0.181	r = 0.571<br>p = 0.041	r = 0.056<br>p = 0.862	r = 0.158<br>p = 0.625	r = 0.227<br>p = 0.502	r = -0.009<br>p = 0.979		
Alpha MC	r = 0.066<br>p = 0.831	r = 0.176<br>p = 0.566	r = -0.246<br>p = 0.440	r = -0.242<br>p = 0.449	r = 0.155<br>p = 0.650	r = 0.473<br>p = 0.142	r = 0.440<br>p = 0.133	

Table 9. All cases without cigarette smoke exposure (n=21)

	TUNEL PC	TUNEL MC	Casp PC	Casp MC	Beta PC	Beta MC	Alpha PC	Alpha MC
TUNEL PC								
TUNEL MC	r = 0.929<br>p < 0.001							
Casp PC	r = 0.042<br>p = 0.856	r = 0.026<br>p = 0.911						
Casp MC	r = 0.033<br>p = 0.886	r = 0.000<br>p = 0.999	r = 0.906<br>p < 0.001					
Beta PC	r = 0.120<br>p = 0.616	r = -0.068<br>p = 0.774	r = -0.247<br>p = 0.294	r = -0.301<br>p = 0.197				
Beta MC	r = -0.235<br>p = 0.328	r = -0.219<br>p = 0.354	r = -0.224<br>p = 0.343	r = -0.256<br>p = 0.276	r = 0.589<br>p = 0.006			
Alpha PC	r = 0.074<br>p = 0.779	r = 0.096<br>p = 0.715	r = -0.119<br>p = 0.648	r = -0.169<br>p = 0.517	r = 0.132<br>p = 0.613	r = -0.243<br>p = 0.348		
Alpha MC	r = -0.0654<br>p = 0.812	r = -0.004<br>p = 0.987	r = -0.121<br>p = 0.656	r = -0.135<br>p = 0.619	r = 0.094<br>p = 0.729	r = 0.003<br>p = 0.991	r = 0.388<br>p = 0.137	

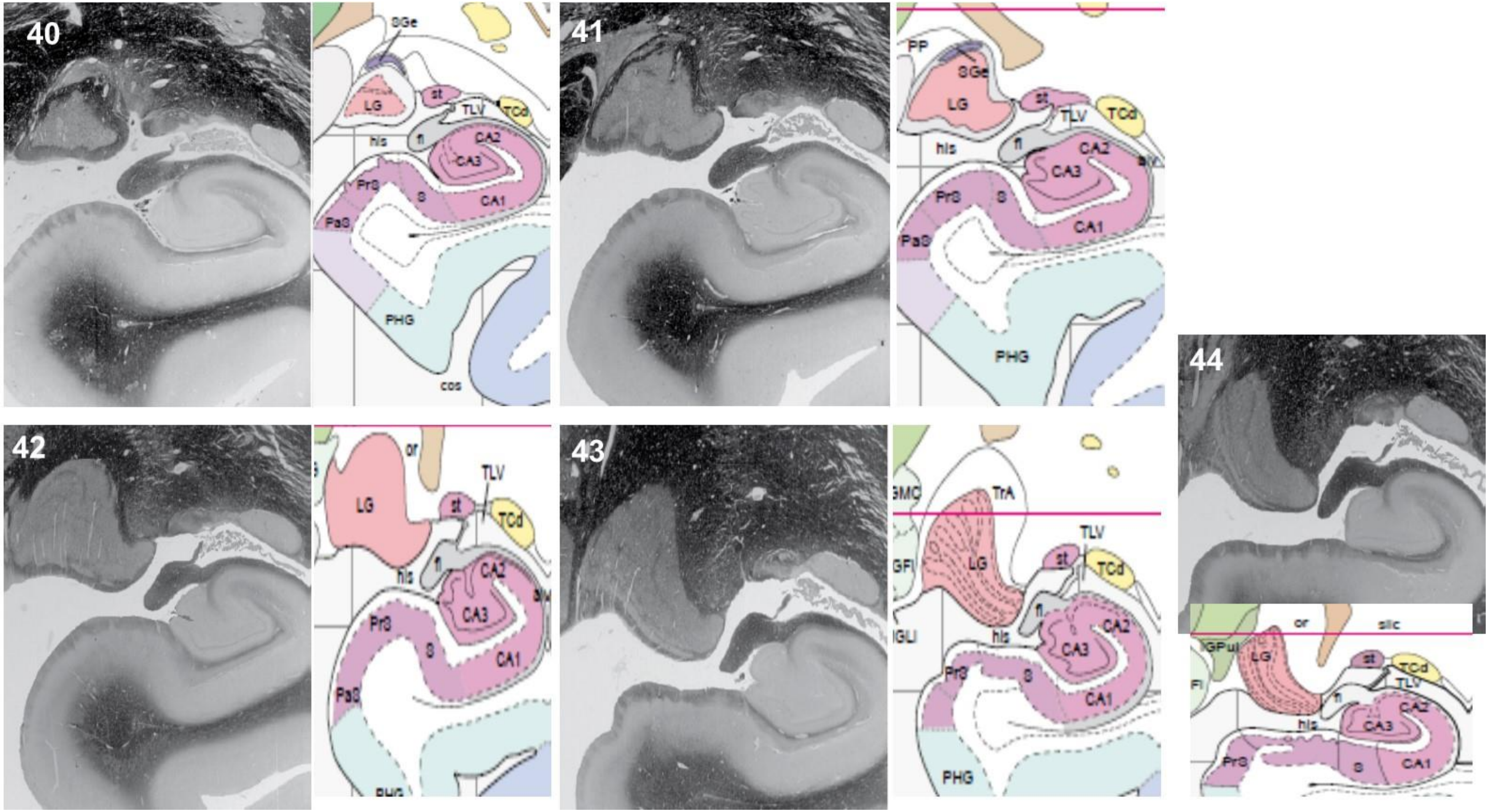

Supplementary Figure 1. Images of the LGN taken from the Atlas of the Human Brain by Mai et al., 1st Edition, 1997, looking from level 40-44 of the "Sections of the Brain". Images available online at https://www.thehumanbrain.info/brain/bn_brain_atlas/brain.html

A

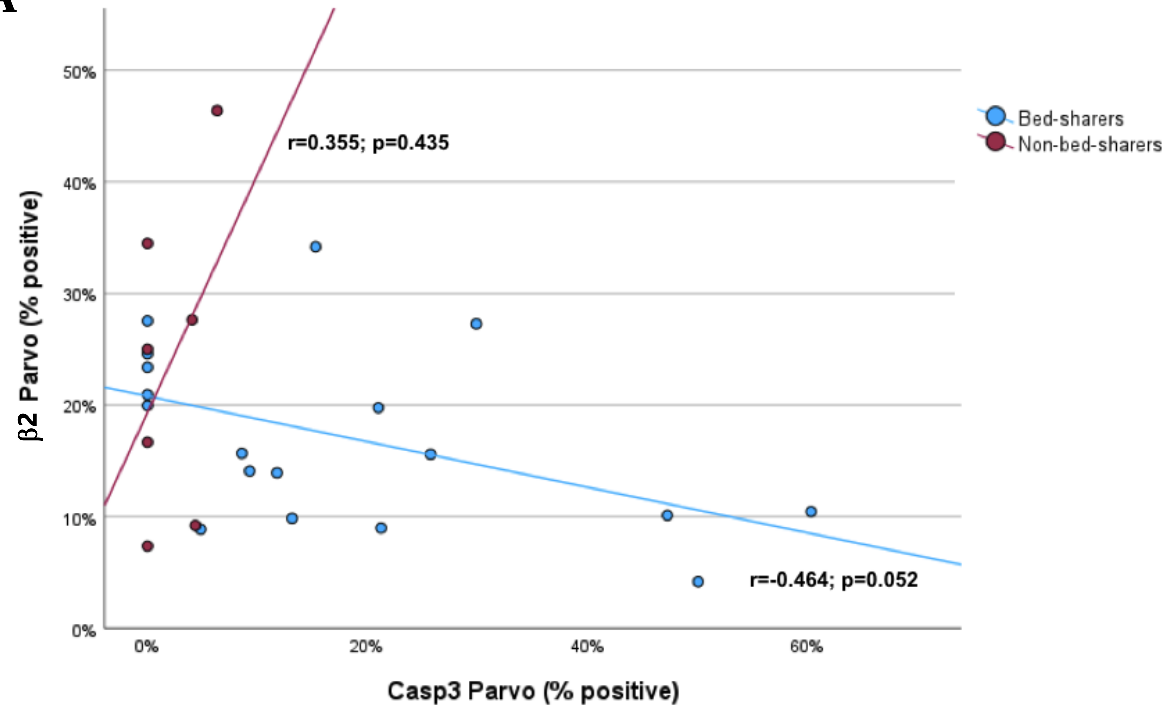

B

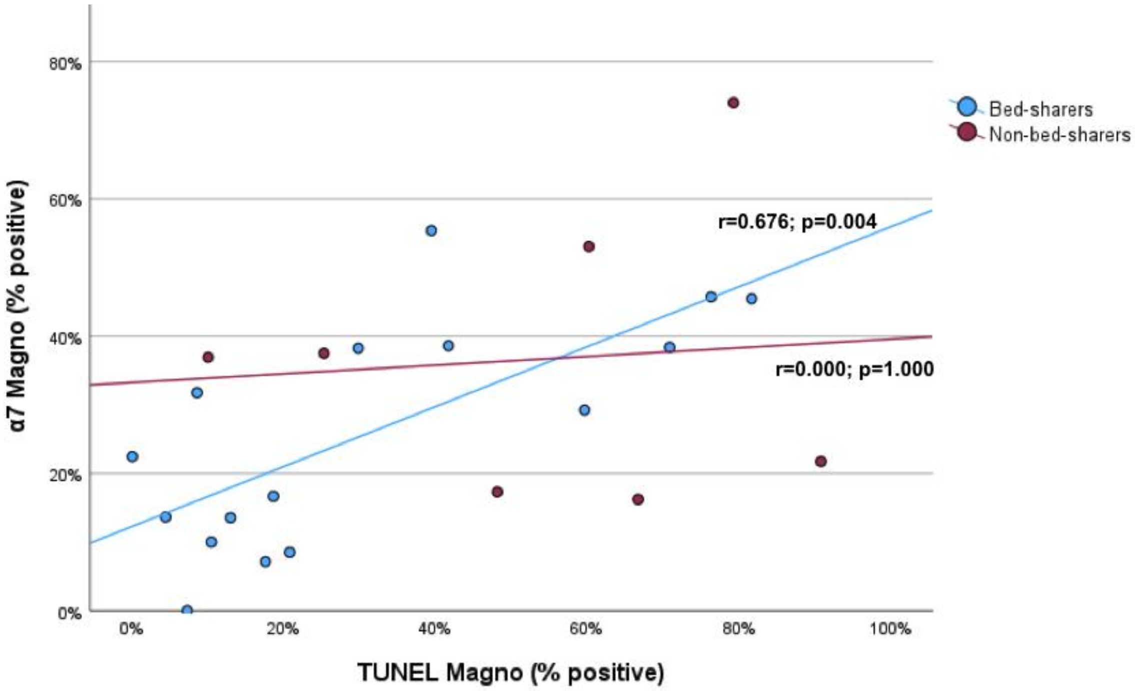

Supplementary Figure 2. Correlation between (A) Casp-3 and β2 nAChR in the PC layer, and (B) TUNEL and α7 nAChR in the MC layer in SIDS II cases when assessing for bed-sharing.
